# Ileocolic intussusception due to a cecal endometriosis: Case report and review of literature

**DOI:** 10.1186/1746-1596-7-62

**Published:** 2012-06-07

**Authors:** Rivkine Emmanuel, Marciano Léa, Polliand Claude, Valenti Antonio, Ziol Marianne, Poncelet Christophe, Barrat Christophe

**Affiliations:** 1 Department of digestive and endocrine surgery, University Hospital Jean Verdier, Assistance Publique-Hôpitaux de Paris, Avenue du 14 Juillet, 93140, Bondy, Paris, France; 2 Department of pathology, University Hospital Jean Verdier, Assistance Publique-Hôpitaux de Paris, Avenue du 14 juillet, 93143, Bondy, France; 3 Department of obstetrics and gynaecology and ART Centre, University Hospital Jean Verdier, Assistance Publique-Hôpitaux de Paris, Avenue du 14 juillet, 93143, Bondy, France

**Keywords:** Endometriosis, Ileocolic intussusception, Digestive endometriosis

## Abstract

**Abstract:**

Cecal endometriosis and ileocolic intussusception due to a cecal endometriosis is extremely rare. We report a case of a woman who presented an ileocecal intussusception due to a cecal endometriosis. The patient gave two months history of chronic periombilical pain requiring regular hospital admission and analgesia. The symptoms were not related to menses. A laparotomy was performed and revealed an ileocolic intussusception. The abdominal exploration did not find any endometriosis lesion. Ileocaecal resection was performed. Microscopic examination showed a cystic component, lined by a regular cylindric epithelium. Foci of endometrial tissu were oberved in the cecal subserosa and muscularis mucosal, with irregular endometrial glands lined by cylindric epithelium without atypia immunostained with CK7, and characteristic endometrial stroma immunostained with CD10. Cecal endometriosis and ileocolic intussusception due to a cecal endometriosis is extremely rare. Diagnose of etiology remains challenging due to the absence of clinical and radiological specific characteristics.

**Virtual slide:**

The virtual slide(s) for this article can be found here: http://www.diagnosticpathology.diagnomx.eu/vs/2975867306869166

## Background

Endometriosis is an estrogen-dependent inflammatory disease that affects 5 to 10% of women of reproductive age in the United States [1]. It is characterised by the presence of endometriosis tissue outside the uterine cavity. Endometriosis is usually confined to the pelvic and reproductive organs but has been described in several remote site including omentum, gastrointestinal tract (rectosigmoid, appendix, small bowel, right colon), umbilicus, lungs, kidney, pancreas and liver [2-6]. Cecal endometriosis and ileocolic intussusception due to a cecal endometriosis is extremely rare. We report a case of a woman who presented an ileocecal intussusception due to a cecal endometriosis.

## Case presentation

A 19 years old multiparous woman was referred to our unit to investigate an abdominal mass. Her last menstrual period was two weeks before. The patient gave two months history of chronic periombilical pain requiring regular hospital admission and analgesia. The symptoms were not related to menses. There were no other significant symptoms. She had a history of endometriosis involving ovaries, and had undergone an ovarian cystectomy for endometrioma one year previously.

The physical exam showed a periombilical palpable mass, painful, and no lymphadenopathy was noted. Complete blood count and biochemical tests were normal.

**Figure 1:**
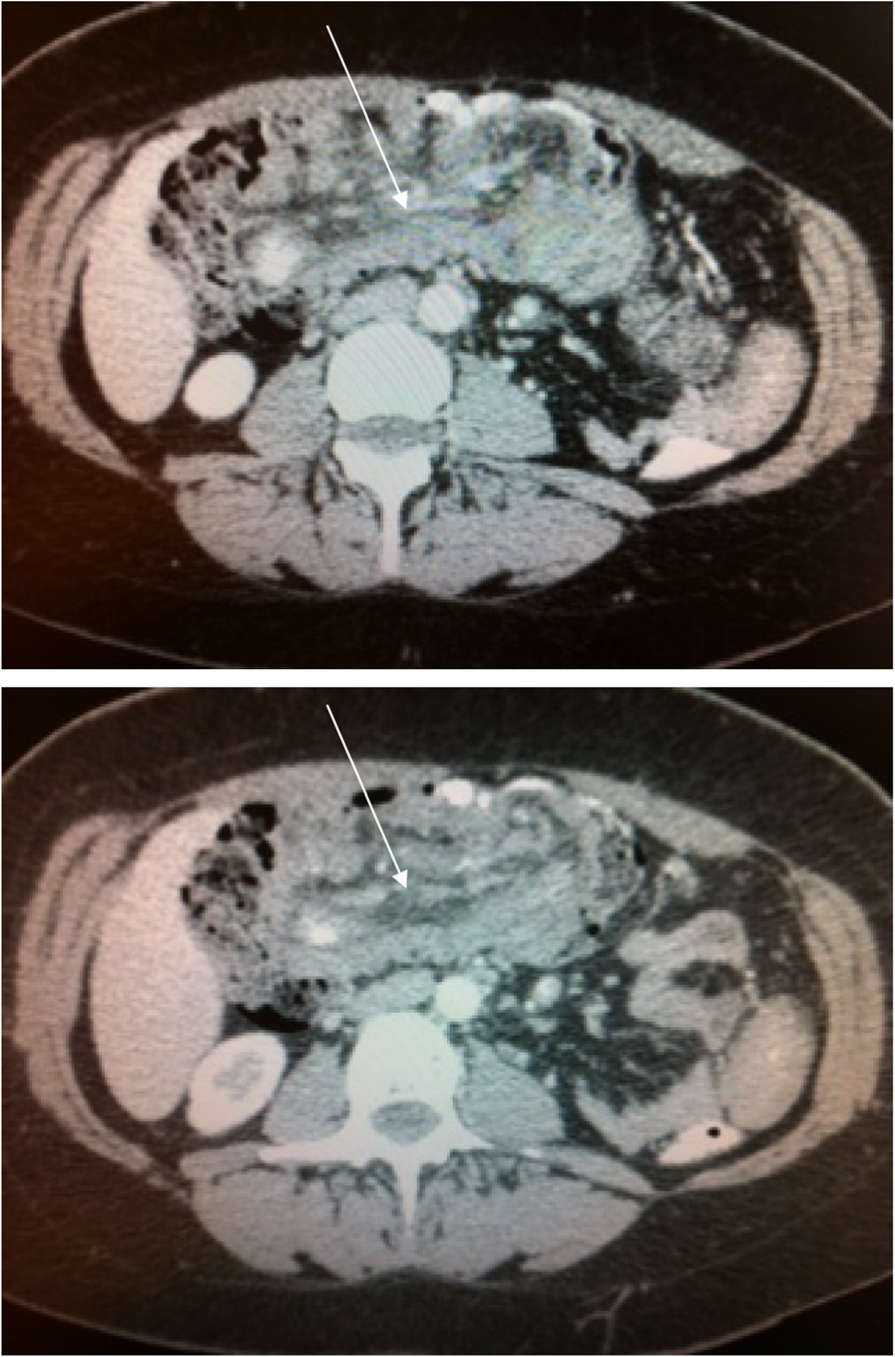
The computed tomography scan shows one an ileocolocolic intussusception.

The computed tomography (Figure 1) scan showed one an ileocolic intussusception. The preoperative diagnoses were a malignant tumour of the colon caecum (adenocarcinoma, sarcoma), or benign tumour (lipoma, villous tumour).

A laparotomy was performed and revealed an ileocolic intussusception. The abdominal exploration did not find any endometriosis lesion. Ileocaecal resection was performed.

Postoperative courses were uneventful. After six months follow-up, patient was asymptomatic.

Macroscopic examination showed a unilocular cystic mass, 5.5*4*4 cm large, which seemed to be developped from subserosa, with mucosal ulceration at the top and haemorragic content.

Microscopic examination showed a cystic component, lined by a regular cylindric epithelium.

**Figure 2:**
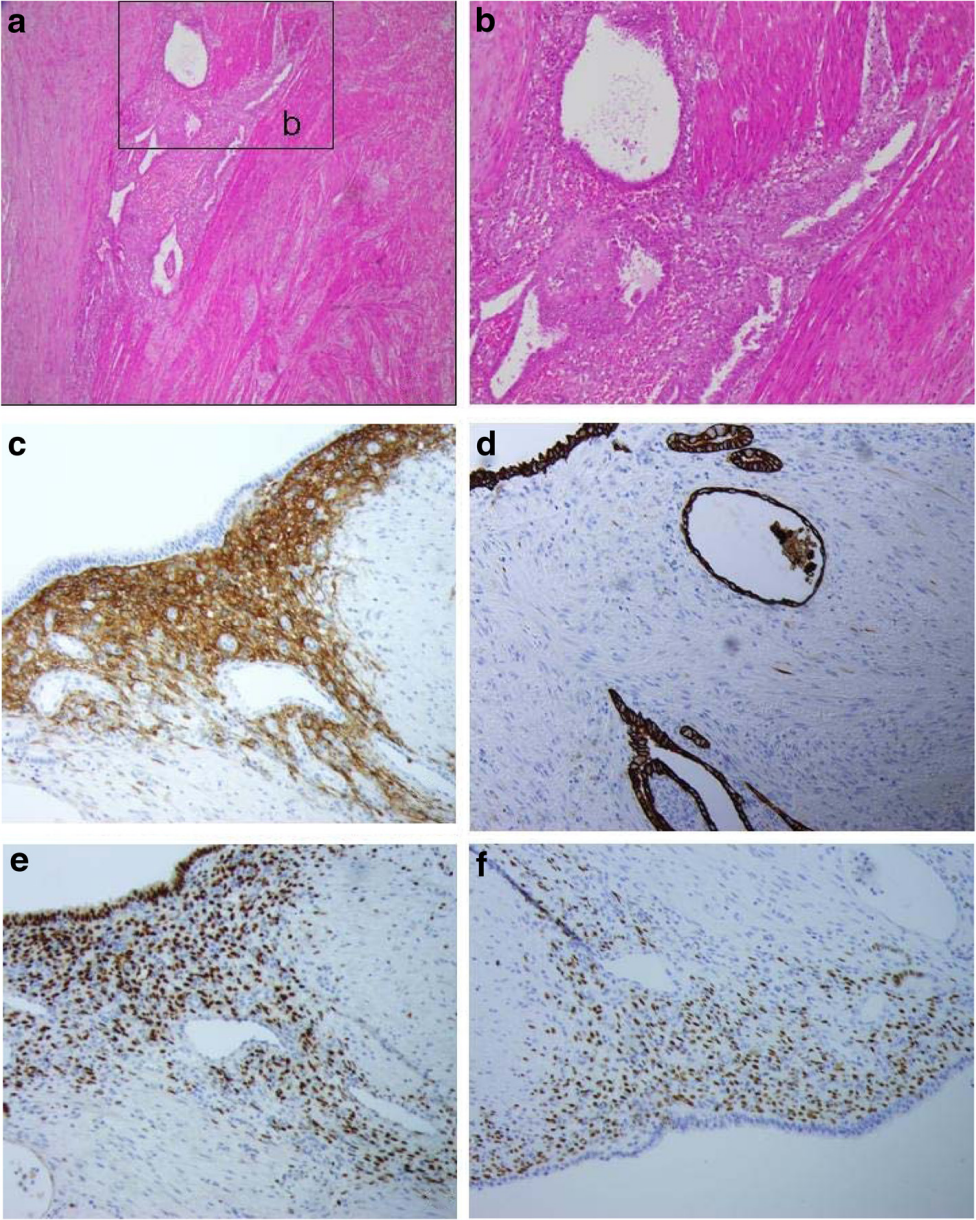
**At microscopic examination, we observed an endometrial tissue developped in the cecal subserosa and muscularis mucosa(a), with irregular endometrial glands (b), and characteristic stroma positive for CD10 immunostaining (c).** Epithelial cells are positive for CK7**(d)**. Moreover, endometrial and stromal cells expressed both estrogen receptors **(e)** and progestative receptors **(f)**.

Foci of endometrial tissu were oberved in the cecal subserosa and muscularis mucosal, with irregular endometrial glands lined by cylindric epithelium without atypia immunostained with CK7, and characteristic endometrial stroma immunostained with CD10 (Figure 2). Stroma and epithelial cells expressed estrogen and progestative receptors. No epithelial hyperplasia, atypia, or invasive carcinoma was observed. These lesions are lined by mucosal ulceration at the top, and surrounded by granulation tissue. We concluded to a cecal endometrioma involving subserosa and muscularis mucosa without malignant transformation.

## Discussion

Endometriosis is an estrogen-dependent disease that affects 5 to 10% of women of reproductive age in the United States [1]. Its defining feature is the presence of ectopic endometrial tissue. The main clinical features are chronic pelvic pain, pain during intercourse, and infertility. Endometriosis can be the result of diverse anatomical or biochemical aberrations of uterine function. The pathogenesis of endometriosis is still unknown. The gold standard for diagnosis of pelvic disease is surgical assessment [1][7].

Endometriosis has been more likely described in pelvic organs (ovaries, fallopian tubes, uterosacral ligaments, Douglas Pouch). It has been described in every part of the body (heart, lung, kidney, gastrointestinal tract, diaphragm, legs, bone, incisional scar, ombilicus, liver), [8][9] except the spleen [3].

Intussusception is defined as the telescoping of a segment of the gastrointestinal tract into an adjacent one. Intussusception is uncommon in adults compared with the pediatric population. It is estimated that only 5% of all intussusceptions occur in adults and approximately 5% of bowel obstructions in adults are the result of intussusception [10]. Leon K. shows in an institutional review of intussusception in adults a pathologic cause identified in 85% of patients with 8 of 22 (36%) small bowel and 4 of 5 (80%) of large bowel lesions being malignant. All small bowel cancers represented metastatic disease and all large bowel malignancies were primary adenocarcinomas [11]. Prystowsky JB shows 1573 consecutive patients with endometriosis diagnosed at laparoscopy or laparotomy, 85 patients (5.4%) had gastrointestinal involvement [12]. Frequently, intestinal localisations of endometriosis are the rectum or the sigmoid, and more rarely appendix, ileum, and right colon [13].

In the literature, only six cases of ileocolic intussusceptions due to a cecal endometriosis were reported [14-19]. We report the seventh case. Aronchick et al. [16], the first case, report a clinical presentation of ileocolic intussusception and digestive hemorrhage. Twenty years later, Denève et al. [15] report the case of a 43-year-old woman, who presented a complete and non-reductible ileo-cecal intussusception with occlusion. Le Meaux et al. [14] report a 40-years-old woman who had an ileo-caeco-colic intussusception on a digestive endometriosis. Koutsourelakiss et al. [18] report a 32-year-old nulliparous Caucasian woman who presented to the emergency department for abdominal pain, distension with nausea and vomiting corresponding to a cecal endometriosis. Maltz et al. [17], show a lesion, with the appearance of inflammatory (Crohn's disease) or infectious (tuberculosis). Indraccolo et al. [19] report a patient who presented an ileocolic intussusception with right iliac fossa pain, distension and diarrhea coverage for laparoscopic debulking of severe endometriosis. In this case, we report another unusual presentation of endometriosis characterized by ileo-cecal intussusception.

The diagnosis of endometriosis may be suspected on the basis of the clinical history. Computed tomography is not the primary imaging for evaluation of digestive endometriosis. However, multislice computed tomography enteroclysis identifies 94.8% of bowel endometriotic nodules [20], and magnetic resonance imaging has a high sensitivity (77%-93%) in the diagnosis of bowel endometriosis [21].

CA-125 is the principal serum marker used in the diagnosis and management of late-stage endometriosis. Cancer antigen CA-125 has been used to monitor the progress of endometriosis [22].

Surgical treatment is indicated for pain, bleeding, and intestinal obstruction. The treatment of small bowel endometriosis is surgical resection of the involved segment, while medical therapy is only a temporary treatment [23].

## Conclusion

Cecal endometriosis and ileocolic intussusception due to a cecal endometriosis is extremely rare. Diagnose of etiology remains challenging due to the absence of clinical and radiological specific characteristics, especially when the symptoms are not related to menses. This disease can be life-threatening, requiring urgent surgery. Digestive endometriosis is established after surgery. The gold standard for diagnosis of pelvic disease is surgical assessment but this treatment can not prevent recurrence. Endometriosis had to be considered in the differential diagnosis of ileocolic intussusception, particularly in patients with known endometriosis.

## Consent

Written informed consent was obtained from the patient for publication of this Case Report and any accompanying images. A copy of the written consent is available for review by the Editor-in-Chief of this journal.

## Competing interests

The authors declare that they have no competing interest.

## Authors' contributions

ER wrote the manuscript. CPon. and CB participated in drafting the manuscript and literature review. ER, CPol. and AV were responsible for acquisition of clinical data, follow-up information and the surgery. LM and MZ participated in making the histopathological diagnosis, conception of the idea and revising the manuscript. All authors have read and approved the final manuscript.
