# Correction to: Ileocolic intussusception due to a cecal endometriosis: Case report and review of literature

**DOI:** 10.1186/s13000-018-0723-y

**Published:** 2018-08-24

**Authors:** Emmanuel Rivkine, Léa Marciano, Claude Polliand, Antonio Valenti, Marianne Ziol, Christophe Poncelet, Christophe Barrat

**Affiliations:** 1 Department of digestive and endocrine surgery, University Hospital Jean Verdier, Assistance Publique-Hôpitaux de Paris, Avenue du 14 Juillet, 93140 Bondy, Paris France; 2 Department of pathology, University Hospital Jean Verdier, Assistance Publique-Hôpitaux de Paris, Avenue du 14 juillet, 93143 Bondy, France; 3 Department of obstetrics and gynaecology and ART Centre, University Hospital Jean Verdier, Assistance Publique-Hôpitaux de Paris, Avenue du 14 juillet, 93143 Bondy, France

## Correction

After publication of this work [1], the authors noticed that the first names and last names of all the authors were inverted. In the original manuscript, they appear on PubMed as:

Emmanuel R, Léa M, Claude P, Antonio V, Marianne Z, Christophe P, Christophe B.

But they should appear as:

Rivkine E, Marciano L, Polliand C, Valenti A, Ziol M, Poncelet C, Barrat C.
